# Supplementary material for: Bottle-grade polyethylene furanoate from ring-opening polymerisation of cyclic oligomers
Source: Nat Commun. 2018 Jul 24;9:2701. doi: 10.1038/s41467-018-05147-y (PMC6057881; doi:10.1038/s41467-018-05147-y)
Supplement: Supplementary file 1 — Supplementary Information [file 41467_2018_5147_MOESM1_ESM.pdf]

Supplementary Information for

Bottle-grade Polyethylene Furanoate from Ring-Opening Polymerisation of Cyclic Oligomers

Rosenboom *et al.*

Institute for Chemical and Bioengineering, ETH Zurich, 8093 Zurich, Switzerland

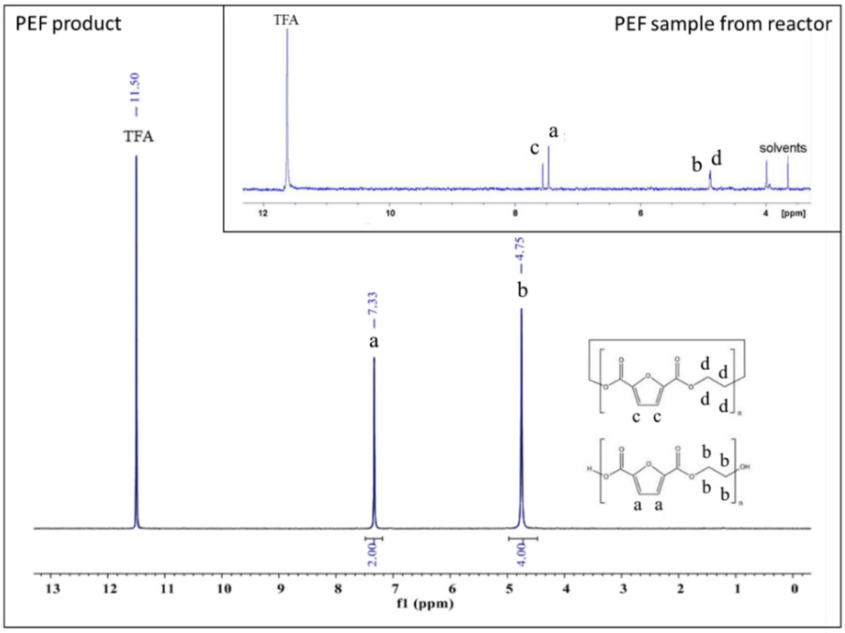

Supplementary Figure 1. Representative ^{1}H NMR spectrum (400 MHz) of PEF product, and PEF in a sample with unreacted cyOEF species from the ROP reactor, measured in TFA-*d* at room temperature.

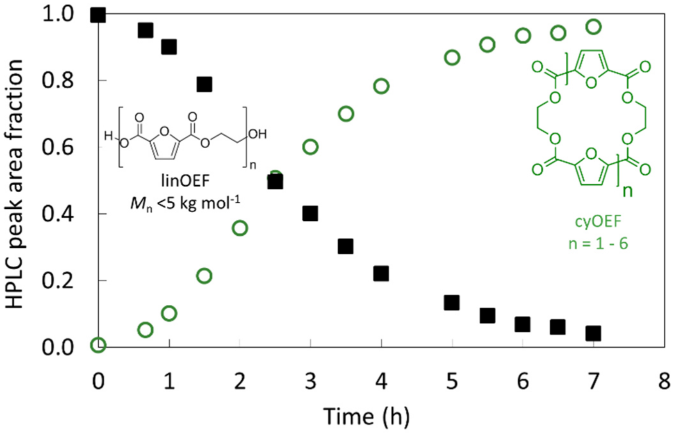

Supplementary Figure 2. Depolymerisation of linear PEF prepolymer "linOEF" (■, sum of all HPLC area associated with linear PEF oligomers) in o-dichlorobenzene at 10 g L^{-1} dilution to the cyclic PEF oligomers "cyOEF" (○, sum of all HPLC area associated with cyclic PEF oligomers).

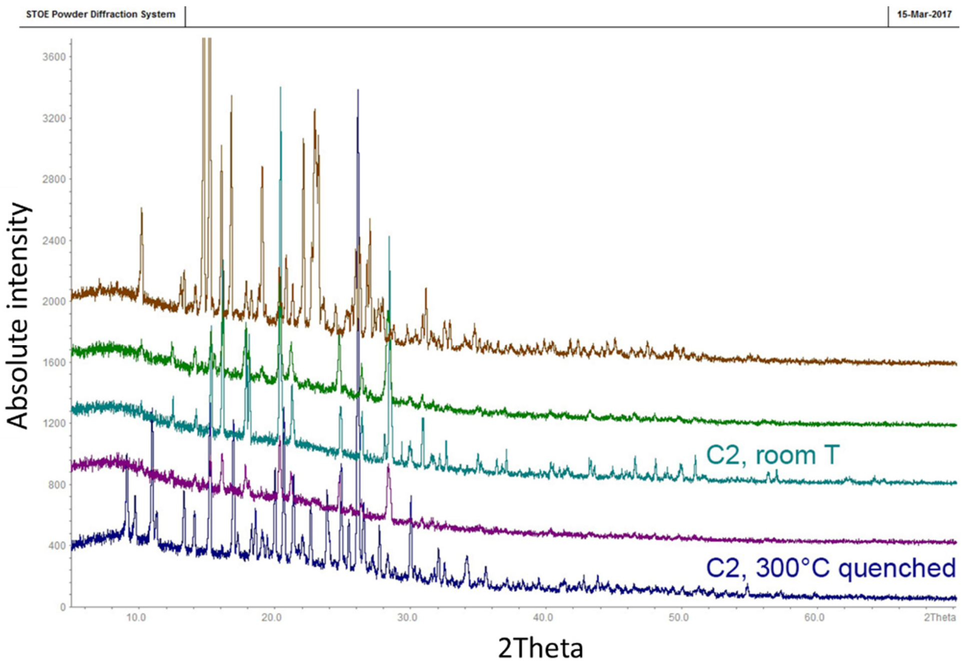

Supplementary Figure 3. Different X-ray powder diffraction patterns of several cyOEF samples, among which C2 at room temperature, and C2 heated to 300°C followed by quenching, proving the occurrence of recrystallisation.

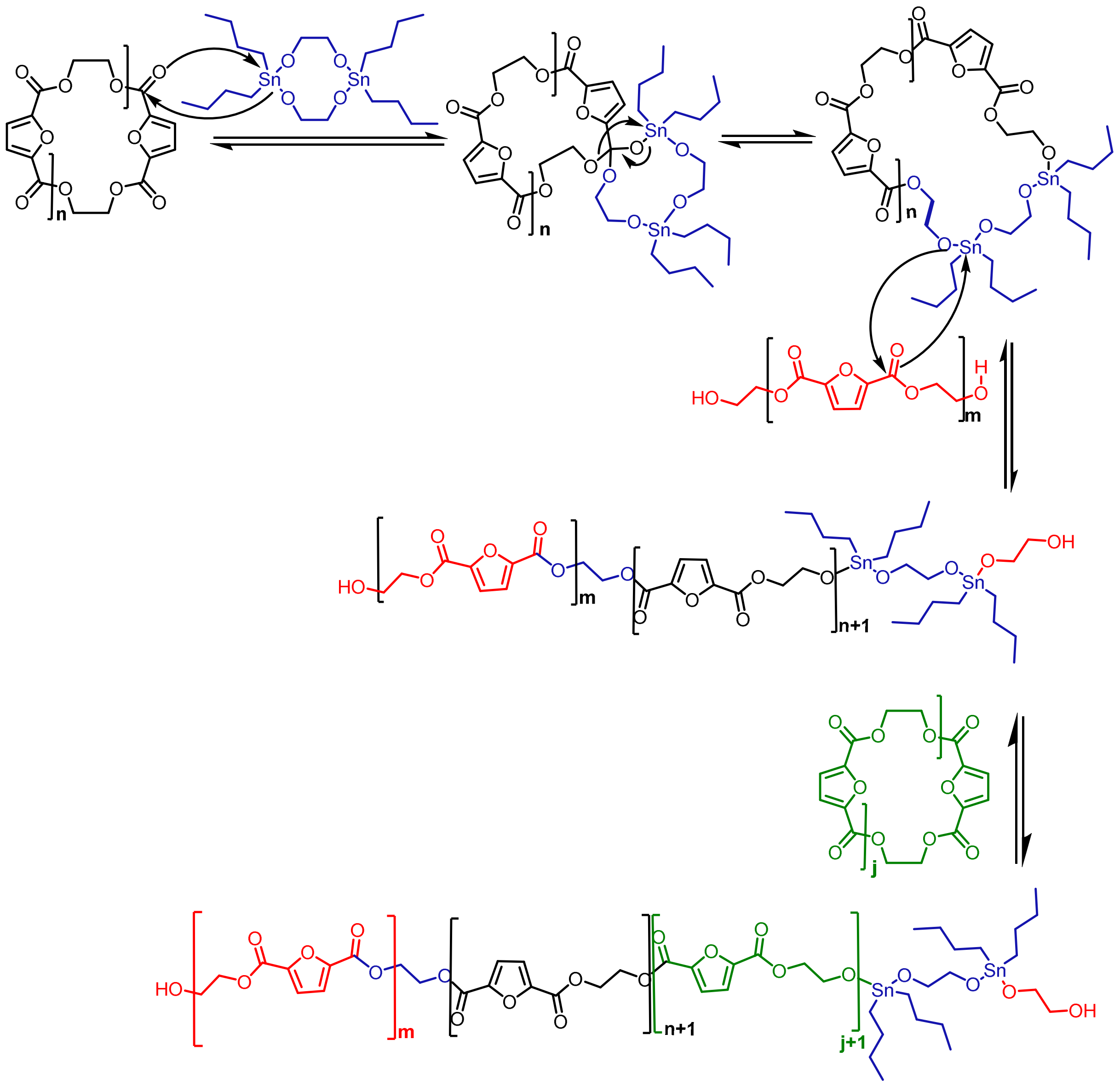

Supplementary Figure 4. Initiator coordination, ring-opening and propagation in cyclic stannoxane (cySTOX)-based ROP to synthesise PEF from cyclic oligomers.

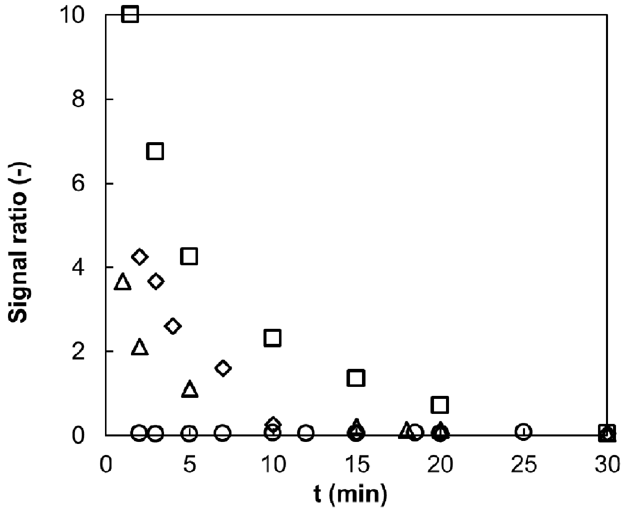

Supplementary Figure 5. The plasticiser tetraglyme at contents of 0% (○), 25% (△), 33% (◇), 100% (□) leaving the ROP reaction due to evaporation. Reaction conditions were 280°C, using 99% pure cyOEF with 0.1% cySTOX initiator in all cases. The abscissa shows the RI signal ratio as the tetraglyme peak signal compared with that of PEF.

Supplementary Table 1. ROP results using another tin-based initiator, $Sn(Oct)_2$ with a 15-fold excess of dodecanol as activator.

**cyOEF purity**	**Plasticisation**	**Initiator**	***T*** (ºC)	**time** (min)	***X*** (%)	M_n (kg mol^{-1})	M_w	**Colour**
99%	33% tg.	0.10% $Sn(Oct)_2$	280	90	95	10.0	20.6	brown
99%	33% tg.	0.20% $Sn(Oct)_2$	280	20	95	12.2	20.7	beige

Supplementary Table 2. ROP results using plasticiser only and a lower amount of cySTOX.

**cyOEF purity**	**Plasticisation**	**Initiator**	***T*** (ºC)	**time** (min)	***X*** (%)	$\boldsymbol{M_n}$ (kg mol^{-1})	$\boldsymbol{M_w}$	**Colour**
99%	40% tg.	-	280	240	74	26.3	46.9	beige
99%	40% tg.	0.05% cySTOX	260	45	65	33.0	69.7	beige

Supplementary Table 3. Molecular weight analysis of PEF and PET from different analytical methods: Absolute molecular weights from SEC coupled with multi-angle light scattering (MALS), diffusion (DOSY) NMR analysis, and molecular weights relative to PMMA standards.

	**M_n (kg mol^{-1})**			**M_w (kg mol^{-1})**		
	**SEC-MALS**	**DOSY NMR**	**SEC (PMMA)**	**SEC-MALS**	**DOSY NMR**	**SEC (PMMA)**
**PEF from ROP**						
1	17.9	17.5	24.1	32.7	33.9	58.0
2	22.3	21.4	37.5	39.9	41.5	79.9
3	32.2	31.8	50.1	61.1	61.6	128.2
4	37.9	37.3	58.9	73.9	72.3	154.6
**PET standards**						
PET standard (Spec: M_w = 38 kg mol^{-1})	20.5	19.5	20.0	37.8	38.5	43.7
PET standard (Spec: M_w = 51 kg mol^{-1})	28.5	26.6	26.4	51.9	52.3	63.7
PET standard Spec: M_w = 74 kg mol^{-1})	37.2	39.6	34.7	73.7	77.3	103.0

Supplementary Table 4. Tensile testing of native PEF compared with bottle-grade commercial PET. Molecular weights after compression molding of films were M_n = 19.9 kg mol^{-1} and M_w = 34.4 kg mol^{-1} for PEF, and M_n = 34.1 kg mol^{-1} and M_w = 60.6 kg mol^{-1} for commercial PET, respectively. Repetitions per strain rate were at least 3.

	**Strain rate** (mm min^{-1})	**Young's Modulus** (MPa)	**Tensile Strength** (MPa)	**Strain at break** (%)
PEF	10	1833.7 ± 67.9	76.4 ± 4.3	4.9 ± 0.3
	1	1893.6 ± 38.3	76.9 ± 5.9	4.9 ± 0.6
PET	10	1098.3 ± 77.4	50.1 ± 2.5	387.9 ± 15.8
	1	1101.9 ± 46.4	47.3 ± 11.7	556.2 ± 161.6
